# Neutrophil-lymphocyte ratio as a predictor of oncologic outcomes in stage IVB, persistent, or recurrent cervical cancer patients treated by chemotherapy

**DOI:** 10.1186/s12885-019-5269-1

**Published:** 2019-01-10

**Authors:** Pornprom Ittiamornlert, Irene Ruengkhachorn

**Affiliations:** Division of Gynecologic Oncology, Department of Obstetrics and Gynecology, Faculty of Medicine Siriraj Hospital, Mahidol University, 2 Wanglang Road, Siriraj, Bangkoknoi, Bangkok, 10700 Thailand

**Keywords:** Cervix, Cancer, Chemotherapy, Blood components, Oncologic outcomes

## Abstract

**Background:**

Cervical cancer patients with stage IVB, persistent, or recurrent disease after complete primary treatment are usually treated with systemic chemotherapy. Circulating blood components have been a target of study relative to their ability to predict cancer outcomes; however, no previous study has focused on patients with advanced, persistent, or recurrent cervical carcinoma who were treated by chemotherapy, which adversely affects hematopoietic and immune activity. The predictive value of complete blood cell differential counts in patients with stage IVB, persistent, or recurrent cervical cancer treated by chemotherapy, may be able to triage these patients.

**Methods:**

This retrospective chart review was conducted in cervical cancer patients with stage IVB disease, persistent disease, or recurrent disease who were treated by chemotherapy during January 2006 to January 2017 were reviewed**.** Follow-up data were collected through July 2017.

**Results:**

A total of 355 cervical carcinoma patients were included. Of those, 63 patients received chemotherapy as primary treatment, and 292 patients received chemotherapy for persistent or recurrent disease. Mean age was 52.5 ± 10.3 years, median age was 51.9 years (IQR: 45.0–59.7), and mean BMI was 23.3 ± 4.9 kg/m^2^. Overall response rate was 37.5%, with a median progression free survival (PFS) of 5.7 months, and with a median overall survival (OS) of 38.1 months. Multivariate analysis revealed elevated platelet count (> 400,000/mm^3^), squamous cell carcinoma subtype, and distant metastasis to be associated with poorer PFS. Elevated neutrophil count (> 7000/mm^3^), elevated platelet count (> 400,000/mm^3^), squamous cell carcinoma subtype, and distant metastasis were found to be associated with poorer OS. Neutrophil-lymphocyte ratio ≥ 3.6 was the most valuable predictor of poor oncologic outcome relative to overall response rate (odds ratio = 1.642, 95% confidence interval [CI]: 1.048–2.572, *P* = 0.030), PFS (hazard ratio [HR] = 1.676, 95% CI: 1.334–2.107, *P* < 0.001), and OS (HR = 2.544, 95% CI: 1.672–3.870, *P* < 0.001).

**Conclusions:**

Neutrophil-lymphocyte ratio ≥ 3.6 was identified as an independent predictor of poor oncologic outcome relative to overall response rate, PFS and OS.

## Background

Cervical cancer is the fourth most common cancer among women worldwide, with an estimated 569,847 new cases and 311,365 deaths in 2018 [1]. In Thailand, cervical cancer is the second most common cancer in women after breast cancer, with approximately 5513 new cervical cancer cases diagnosed in 2014 [2]. Stage I-IIA cervical cancer is usually curable with primary treatment, either by surgery or chemoradiotherapy. Chemoradiotherapy is also used as a primary treatment in stage IB-IVA disease. However, patients who have stage IVB, persistent, or recurrent disease after complete primary treatment are usually treated with systemic chemotherapy [3].

Oncologic outcomes after chemotherapy were fair, with overall response rates that varied from 22 to 35% [4–6]. Multiple factors, including stage of disease, lymph node metastasis, and various biological markers, have been studied in order to identify significant prognostic factors that predict cancer specific survival. Circulating blood components have also been a target of study relative to their ability to predict cancer outcomes. Association between tumors and inflammatory response has been reported in a variety of cancers [7, 8]. However, the findings of those studies varied according to the specific organ being studied. Many aspects of the immune system have been studied in cervical cancer patients; however, all of those studies were conducted in a surgical setting, all were stage-specific, or all patients had squamous cell carcinoma subtype [9–12]. Based on our review of the literature, no previous study has focused on patients with advanced, persistent, or recurrent cervical carcinoma who were treated by chemotherapy, which directly destroys hematopoietic activity and may cause differences in immune activity. Accordingly, the aim of this study was to investigate the predictive value of complete blood cell differential counts relative to overall response rate, progression free survival (PFS), and overall survival (OS) in patients with stage IVB, persistent, or recurrent cervical cancer treated by chemotherapy.

## Methods

### Participants and design

**Fig. 1:**
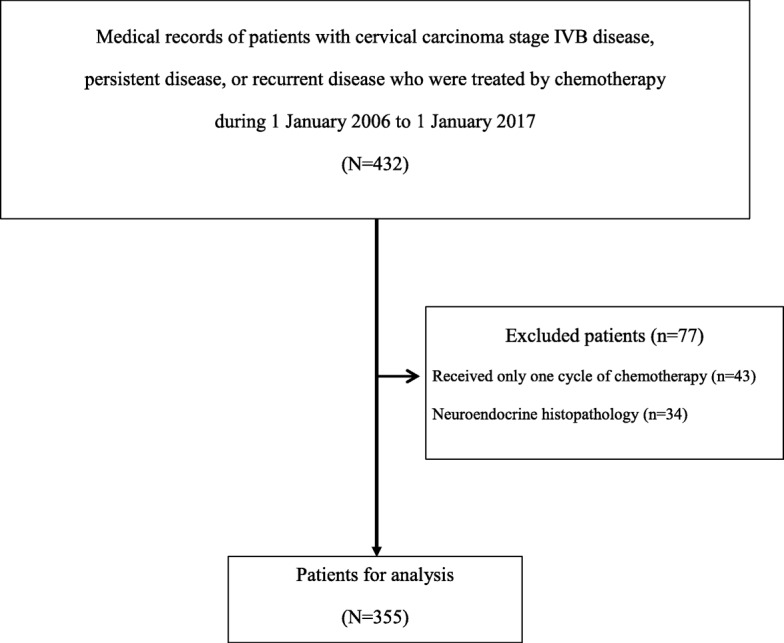
Flowchart describing the patient enrollment process

After receiving ethical approval from the Siriraj Institutional Review Board (SIRB) (COA no. Si 025/2017), a retrospective chart review was conducted in cervical cancer patients with stage IVB disease, persistent disease, or recurrent disease who were treated by chemotherapy during 1 January 2006 to 1 January 2017 at the Division of Gynecologic Oncology, Department of Obstetrics and Gynecology, Faculty of Medicine Siriraj Hospital, Mahidol University, Bangkok, Thailand. Siriraj Hospital is Thailand’s largest national tertiary referral center. Patients that received only one cycle of chemotherapy, that had neuroendocrine histopathology, or that had other disease that affects blood component levels (e.g., hematologic malignancies, infectious disease, or autoimmune disease) were excluded (Fig. 1).

### Measures

Data gathered from medical records included demographic data, tumor characteristics, primary treatment data, chemotherapeutic agents, baseline complete blood count values at initiation of the first cycle of chemotherapy, and oncologic outcomes. Demographic, anthropometric, and other clinical data included age, body mass index (BMI), menopausal status, parity, underlying diseases, and presenting symptoms. BMI was stratified according to World Health Organization (WHO) recommendations with modification into 3 groups (< 18.5 = underweight, 18.5 to 24.9 = normal BMI, and ≥ 25 = overweight or obesity). Tumor characteristics included gross appearance, histopathology types, the International Federation of Gynecology and Obstetrics (FIGO) stage, primary treatment, and sites of disease before chemotherapy initiation. Oncologic outcomes relative to overall response rate (defined as rate of complete response plus rate of partial response), PFS, and OS were evaluated. Cancer stage was reclassified according to FIGO 2018 guidelines [3]. Complete blood cell counts and automated differential counts were performed within 1 week before initiation of the first cycle of chemotherapy. The normal level of each hematologic component was determined by Siriraj hospital’s hematologic laboratory. Since there is no established neutrophil-lymphocyte ratio cut-point, the median neutrophil-lymphocyte ratio of 3.6 that was identified in this study was used as the cut-point in this study. Treatment response was reclassified according to the Response Evaluation Criteria in Solid Tumor (RECIST) 1.1 guideline [13]. Overall response rate was defined as rate of complete response plus rate of partial response. Cervical carcinoma that reappeared at ≥6 months after complete primary treatment was defined as recurrent disease. Disease that persisted or reappeared within 6 months after primary treatment was defined as persistent disease. PFS was defined as time from initiation of chemotherapy to reappearance or progression of disease. OS was defined as time from initiation of chemotherapy to cancer specific death.

### Statistical analysis

The sample size was calculated based on a 30% response rate to chemotherapy. This rate was derived from the reported findings from 3 previous studies that reported response rates that ranged from 22 to 35% [4–6]. In order to achieve a level of confidence of at least 95%, a minimum sample size of 355 patients was required. SPSS version 18.0 (SPSS, Inc., Chicago, IL, USA) was used for statistical analysis. Kolmogorov-Smirnov test was used to test the normality of continuous data. Demographic data were summarized using descriptive statistics. Data are presented as number and percentage, mean ± standard deviation (SD), or median and interquartile range (IQR). Univariate analysis was performed to individually evaluate the predictive significance of each factor. All risk factors with a *p*-value of less than 0.05 in univariate analysis were included in multiple logistic regression analysis to identify independent predictors for overall response rate, PFS, and OS. Survival analysis was performed using Kaplan-Meier method and Cox proportional hazard regression analysis. A *p*-value of < 0.05 was considered to be statistically significant for all tests.

## Results

A total of 355 cervical carcinoma patients were analyzed. Of those, 63 patients received chemotherapy as primary treatment, and 292 patients received chemotherapy for persistent or recurrent disease. Mean age was 52.5 ± 10.3 years, median age was 51.9 years (IQR = 45.0–59.7), and mean BMI was 23.3 ± 4.9 kg/m^2^. One hundred and sixty-nine women (47.6%) were in menopause. Median parity was 2 (IQR = 1–3), and 71.8% of patients received a platinum-based agent (cisplatin or carboplatin depending on patient renal function) combined with paclitaxel. Median number of chemotherapy cycles was 6 (IQR = 5–6). The median value of hematologic components was, as follows: white blood cells 6900/mm^3^ (IQR = 5380-8500), neutrophils 4833.4/mm^3^ (IQR = 3447.6-6280.4), lymphocytes 1251.6/mm^3^ (IQR = 910.8–1656.7), monocytes 473.1/mm^3^ (IQR = 343.0–564.0), eosinophils 139.4/mm^3^ (IQR = 72.7–260.4), neutrophil-lymphocyte ratio 3.6 (IQR 2.6–6.0), hemoglobin 10.9 g/dL (IQR = 9.8–12.1), and platelets 339,000/mm^3^ (IQR = 266,000-431,000). Clinical and tumor characteristics, primary treatment methods, complete blood cell differential counts, chemotherapeutic regimens, and response evaluation in 355 studied patients are shown in Table 1.

**Table 1:** Clinical and tumor characteristics, therapeutic regimens, and clinical response in 355 cervical cancer patients

Characteristics	Number (%)
Histopathology	
Squamous cell carcinoma	209 (58.8)
Adenocarcinoma	120 (34.0)
Adenosquamous carcinoma	15 (4.1)
Serous carcinoma	1 (0.3)
Clear cell carcinoma	3 (0.8)
Mixed types	1 (0.3)
Undifferentiated carcinoma	5 (1.4)
Adenosarcoma	1 (0.3)
Initial FIGO stage	
IA	6 (1.7)
IB	53 (14.9)
IIA	15 (4.2)
IIB	90 (25.4)
IIIA	1 (0.3)
IIIB	127 (35.8)
IVA	9 (2.5)
IVB	50 (14.1)
Inadvertent hysterectomy	3 (0.8)
No data	1 (0.3)
Primary treatment	
Radiotherapy	42 (11.8)
Concurrent chemoradiotherapy	209 (58.9)
Surgery	42 (11.8)
Chemotherapy	62 (17.5)
Disease sites before chemotherapy initiation	
Locoregional	138 (38.9)
Distant	90 (25.4)
Both	127 (35.8)
Chemotherapeutic agents	
Platinums^a^ + Paclitaxel	255 (71.8)
Platinums^a^ + Fluorouracil based	27 (7.6)
Cisplatin + Topotecan	10 (2.8)
Cisplatin + Mitomycin	13 (3.7)
Cisplatin + Ifosfamide	12 (3.4)
Cisplatin + Gemcitabine	1 (0.3)
Single drugs^b^	37 (10.4)
Response to treatment	
Complete response	79 (22.3)
Partial response	54 (15.2)
Stable of disease	108 (30.4)
Disease progression	114 (32.1)
Recurrent or persistent disease	
No recurrence	35 (9.9)
Recurrence after complete response	44 (12.4)
Persistent disease	276 (77.7)
Last contact outcomes	
Alive without disease	39 (11.0)
Alive with disease	204 (57.5)
Death without disease	0
Death with disease	112 (31.5)

*FIGO* the International Federation of Gynecology and Obstetrics

^a^Platinums include either Cisplatin or Carboplatin

^b^Single agents compose of Cisplatin, Carboplatoin, Mitomycin C, Paclitaxel, Gemcitabine, Ifosfamide

The overall response rate was 37.5% (133/355 patients). Of those 133 patients, 98 patients (73.7%, 95% confidence interval [CI]: 66.1–80.7) had recurrence or progression of disease, and the remaining 35 patients achieved remission after the first-round chemotherapeutic treatment until the end of the study. The median PFS in all study patients was 5.7 months (IQR = 4.3–10.4). Risk of recurrence or progression at 1-year, 2-year, 3-year, and 5-year was 77.6, 87.6, 90.6, and 91.4%, respectively. The median OS was 38.1 months (IQR = 16.8–71.0). The 1-year, 2-year, 3-year, and 5-year OS rate was 81.1, 64.0, 54.0, and 45.0%, respectively.

**Fig. 2:**
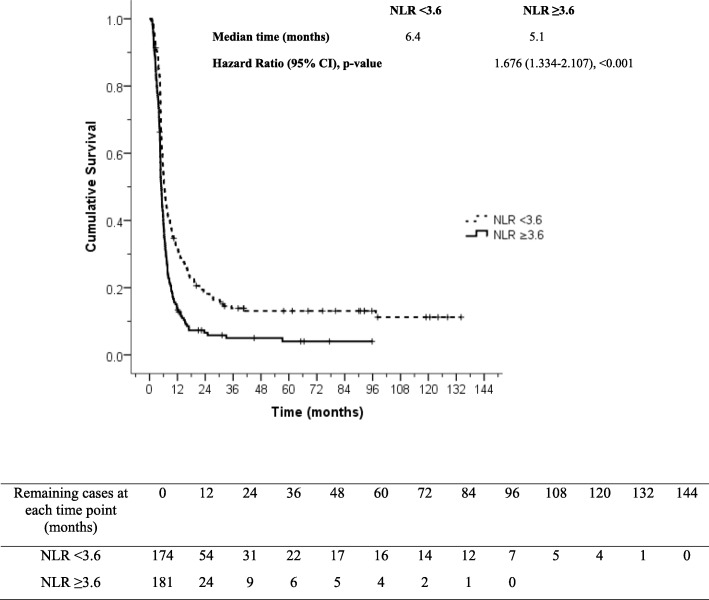
Progression free survival in 355 cervical cancer patients stratified by neutrophil-lymphocyte ratio (NLR), *P* < 0.001

**Fig. 3:**
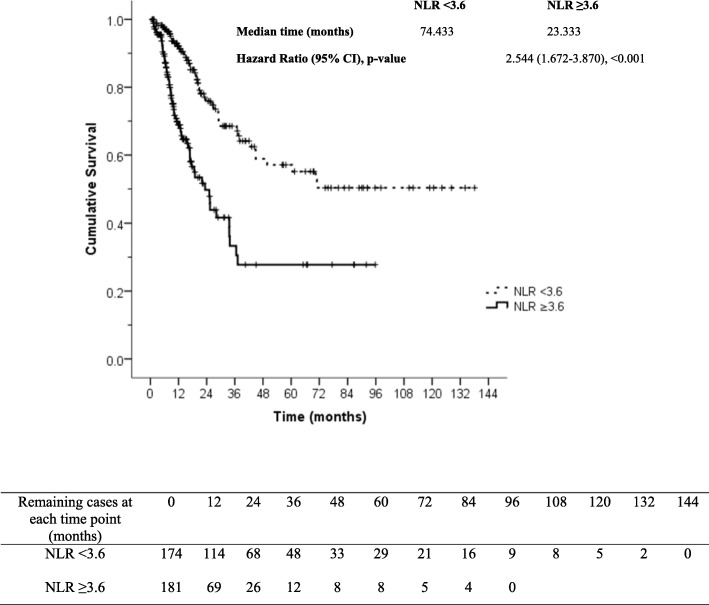
Overall survival in 355 cervical cancer patients stratified by neutrophil-lymphocyte ratio (NLR), *P* < 0.001

Univariate and multivariate analysis for various factors that predict overall response rate, PFS, and OS are presented in Tables 2 and 3, respectively. Multivariate analysis revealed the following: BMI < 25 kg/m^2^ showed association with poor overall response rate; high neutrophil count (> 7000/mm^3^) was identified as a predictor of short OS; and squamous cell carcinoma subtype, distant metastatic disease, and high platelet count (> 400,000/mm^3^) were found to be individually associated with short PFS and OS. The results of significant factors in multivariate analysis were shown in Table 3. The most valuable finding from multivariate analysis was neutrophil-lymphocyte ratio ≥ 3.6 as the only factor independently predictive of poor oncologic outcomes, relative to overall response rate, PFS, and OS. Progression free survival and overall survival curves of 355 study patients stratified by neutrophil-lymphocyte ratio are shown in Figs. 2 and 3, respectively.

**Table 2:** Univariate analysis for factors associated with response rate, progression free survival and overall survival in 355 cervical cancer patients

Variables	Overall response rate		Progression free survival		Overall survival	
	n (%)	*P*	HR (95% CI)	*P*	HR (95% CI)	*P*
Age, years						
< 50 (*n* = 157)	66 (42.0)	0.113	reference		reference	
≥ 50 (*n* = 198)	67 (33.8)		1.091 (0.875–1.362)	0.439	1.050 (0.722–1.527)	0.797
Menopausal status						
Premenopause (*n* = 186)	71 (38.2)	0.773	reference		reference	
Postmenopause (*n* = 169)	62 (36.7)		1.012 (0.812–1.260)	0.917	1.002 (0.691–1.452)	0.994
Body mass index, kg/m^2^						
< 18.5 (*n* = 192)	61 (31.8)	0.003^a^	1.659 (1.160–2.372)	0.003	1.598 (1.035–2.466)	0.034
18.5–24.9 (*n* = 50)	15 (30.0)		1.463 (1.139–1.879)	0.003	2.571 (1.441–4.587)	0.001
≥ 25 (*n* = 113)	57 (50.4)		reference		reference	
Patient group						
Primary (*n* = 63)	22 (34.9)	0.646	reference		reference	
Persistent/recurrent (*n* = 292)	111 (38.0)		0.772 (0.582–1.025)	0.073	0.707 (0.436–1.149)	0.162
Initial FIGO stages (*n* = 351)						
I-II (*n* = 164)	66 (40.2)	0.395	reference		reference	
III-IV (*n* = 187)	67 (35.8)		1.294 (1.037–1.617)	0.023	0.915 (0.629–1.329)	0.640
Histopathology						
Non-SCC (*n* = 146)	60 (41.1)	0.237	reference		reference	
SCC (*n* = 209)	73 (34.9)		1.281 (1.023–1.604)	0.031	1.350 (0.920–1.980)	0.125
Disease site before chemotherapy initiation						
Locoregional (*n* = 138)	56 (40.6)	0.334	reference		reference	
Distant metastasis (*n* = 217)	77 (35.5)		1.353 (1.076–1.702)	0.010	1.971 (1.302–2.983)	0.001
White blood cell count, /mm^3^						
≤ 10,000 (*n* = 300)	118 (39.3)	0.089	reference		reference	
> 10,000 (*n* = 55)	15 (27.3)		1.683 (1.252–2.263)	0.001	2.339 (1.487–3.678)	< 0.001
Neutrophil count, /mm^3^						
≤ 7000 (*n* = 288)	114 (39.6)	0.087	reference		reference	
> 7000 (*n* = 67)	19 (28.4)		1.673 (1.268–2.208)	< 0.001	2.610 (1.706–3.993)	< 0.001
Lymphocyte count, /mm^3^						
≥ 2000 (*n* = 44)	20 (45.5)	0.242	reference		reference	
< 2000 (*n* = 311)	113 (36.3)		1.353 (0.960–1.908)	0.085	1.545 (0.880–2.713)	0.130
NLR						
< 3.6 (*n* = 174)	77 (44.3)	0.010^b^	reference		reference	
≥ 3.6 (*n* = 181)	56 (30.9)		1.648 (1.318–2.060)	< 0.001	2.759 (1.873–4.065)	< 0.001
Monocyte count, /mm^3^						
≤ 970 (*n* = 348)	132 (37.9)	0.263	reference		reference	
> 970 (*n* = 7)	1 (14.3)		3.038 (1.428–6.462)	0.004	3.641 (0.884–14.994)	0.074
Eosinophil count, /mm^3^						
≤ 750 (*n* = 340)	126 (37.1)	0.452	reference		reference	
> 750 (*n* = 15)	7 (46.7)		1.207 (0.706–2.064)	0.491	0.679 (0.215–2.138)	0.508
Hemoglobin, g/dL						
≥ 12 (*n* = 93)	44 (47.3)	0.022^c^	reference		reference	
< 12 (*n* = 262)	89 (34.0)		1.260 (0.982–1.618)	0.069	1.308 (0.853–2.007)	0.219
Platelet count, /mm^3^						
≤ 400,000 (*n* = 246)	95 (38.6)	0.500	reference		reference	
> 400,000 (*n* = 109)	38 (37.5)		1.560 (1.232–1.975)	< 0.001	1.963 (1.327–2.903)	0.001

*HR* hazard ratio, *CI* confidence interval, *FIGO* the International Federation of Gynecology and Obstetrics, *NLR* neutrophil-lymphocyte ratio, *SCC* squamous cell carcinoma

^a^Statistical significance was observed between patients with BMI ≥25 kg/m^2^and BMI 18.5–24.9 kg/m^2^, and between patients with BMI ≥25 kg/m^2^ and BMI < 18.5 kg/m^2^

^b^Odds ratio = 1.772, 95% CI: 1.147–2.736

^c^Odds ratio = 1.745, 95% CI: 1.079–2.823

**Table 3:** Multivariate analysis for independent factors associated with response rate, progression free survival and overall survivals in 355 cervical cancer patients (only significant results were shown)

Variables	Overall response rate		Progression free survival		Overall survival	
	OR (95% CI)	*P*	HR (95% CI)	*P*	HR (95% CI)	*P*
Body mass index, kg/m^2^						
< 18.5	2.064 (1.267–3.364)	0.004	–		–	
18.5–24.9	2.165 (1.051–4.461)	0.036				
≥ 25	reference					
Histopathology						
Non-SCC	–		reference		reference	
SCC			1.334 (1.060–1.678)	0.014	1.533 (1.034–2.272)	0.034
Disease sites before chemotherapy initiation						
Locoregional	–		reference		reference	
Distant metastasis			1.315 (1.042–1.660)	0.021	2.008 (1.319–3.056)	0.001
Neutrophil count, /mm^3^						
≤ 7000	–		–		reference	
> 7000					1.821 (1.139–2.910)	0.012
NLR						
< 3.6	reference		reference		reference	
≥ 3.6	1.642 (1.048–2.572)	0.030	1.676 (1.334–2.107)	< 0.001	2.544 (1.672–3.870)	< 0.001
Platelet count, /mm^3^						
≤ 400,000	–		reference		reference	
> 400,000			1.334 (1.060–1.678)	0.014	1.633 (1.078–2.474)	0.021

*CI* confidence interval, *HR* hazard ratio, *NLR* neutrophil-lymphocyte ratio, *OR* odds ratio, *SCC* squamous cell carcinoma

## Discussion

Chemotherapy is administrated for survival improvement in cervical cancer patients who have metastatic, persistent or recurrent disease. Doublets of platinum-based agents with other agents, such as topotecan, paclitaxel, gemcitabine, vinorelbine, ifosfamide, and 5-fluorouracil, are prescribed in this patient population worldwide [4–6, 14–16]. Our previous clinical study revealed satisfactory results from this treatment strategy, with an overall response rate of 37.8%. In that study, the lone independent clinical predictor of shorter OS was distant metastatic disease, while BMI ≥25 kg/m^2^ and non-squamous cell carcinoma were found to be independent predictors of longer PFS [17]. Previous studies reported a range of response rates and survival times, with an overall response rate that varied from 22.3 to 62.6%. Reported PFS times and OS times ranged from 5.6 to 6.9 months and 11.6 to 18.3 months, respectively. Japanese and Thai studies showed favorable oncologic outcomes compared to outcomes reported in several Gynecologic Oncology Group (GOG) studies [4, 6, 14–17]. Individual host-related factors might be major prognostic indicators related to survival, including BMI, performance status, distant metastasis, and systemic inflammatory response.

Inflammatory cell and immune response play crucial roles in oncogenic transformation, disease progression, and patients’ outcomes. Tumor-associated leukocytosis is a paraneoplastic syndrome that is reported in various types of advanced solid tumor, and that is associated with poor survival in kidney cancer, melanoma, colorectal cancer, gastric and esophageal cancer, hepatocellular carcinoma, cholangiocarcinoma, head and neck cancers, and lung cancer [7]. Neutrophils are the most common leukocyte subset, and they play a pivotal role in cancer-related inflammation. It is, therefore, plausible that neutrophil count might increase during the initial phase of cancer development. The present study revealed elevated neutrophil count to be an independent predicting factor of short OS, which is consistent with the findings of previous studies [7]. Furthermore, impairment of neutrophil migration was observed during the invasive stage of cervical cancer, when compared with healthy women [18].

A high pretreatment neutrophil-lymphocyte ratio was reported to be a poor prognostic factor in solid tumors. Although the mechanisms remain unclear, recent studies reported an elevated neutrophil-lymphocyte ratio to be associated with elevation of cytokines that increased tumor macrophage function, including: interleukin-1 (IL-1), IL-6, IL-7, IL-8, IL-12, IL-17, granulocyte colony-stimulating factor (G-CSF) and monocyte chemoattractant protein-1 [8, 19]. Circulating lymphocytes produce cytokines as a cellular immune response to prevent tumor growth [12]. Increased number of peripheral blood CD8 tumor infiltrating lymphocytes or detection of human papillomavirus specific T-cells are significant positive predictors of treatment outcomes in cervical cancer patients [10]. Lymphopenia at treatment initiation was associated with short survival in solid tumors in lung cancer, breast cancer, pancreatic cancer, colorectal cancer, and locally advanced cervical cancer [20]. Thus, increased peripheral neutrophil and decreased lymphocyte counts reflect enhanced tumor proliferation. High neutrophil-lymphocyte ratio corresponds with poorer outcomes, including more advanced stage, disease progression, metastatic lesions, and short survival [8]. Moreover, high neutrophil-lymphocyte ratio predicted poorer survival, and high lymphocyte count predicted better survival in ovarian cancer patients [21]. There is substantial heterogeneity among the neutrophil-lymphocyte ratio thresholds used to predict patient outcomes (range: > 2 to 5). Some of the reported thresholds were based on normal laboratory values, while others used median values from preliminary studies. Zhang, et al. reported that a preoperative neutrophil-lymphocyte ratio ≥ 2.213 was significantly associated with shorter PFS (HR = 1.799, 95% CI: 1.069–3.028, *P* = 0.027) in patients with clinical stage I-II cervical carcinoma [12]. A Korean study in 1061 patients diagnosed with stage IB-IVA cervical carcinoma found pretreatment neutrophil-lymphocyte ratio ≥ 1.9 to be significantly associated with advanced stage and younger age. The same study found a neutrophil-lymphocyte ratio ≥ 1.9 to be an independent predictor of poor PFS and OS with HR of 1.13 (95% CI: 1.08–1.18, *P* < 0.001), and HR of 1.19 (95% CI: 1.13–1.25, *P* < 0.001), respectively [9]. In a concurrent chemoradiotherapy setting, neutrophil-lymphocyte ratio ≥ 2.5 predicted poor response, shorter PFS (HR = 1.53, 95% CI: 1.19–1.97, *P* = 0.001), and shorter OS (HR = 2.80, 95% CI: 0.83–9.34, *P* = 0.005) [22]. A pretreatment neutrophil-lymphocyte ratio ≥ 3.03 before treatment with radiotherapy with/without chemotherapy was a negative predictive factor for PFS and OS (HR = 3.579, 95% CI: 2.106–6.082, *P* < 0.001, and HR = 3.322, 95% CI: 1.905–5.790, *P* < 0.001, respectively) [23]. Importantly, in the current study, a neutrophil-lymphocyte ratio ≥ 3.6 was found to be an independent predictor of treatment failure and short survival in cervical cancer patients who received chemotherapy. Taken together, the aforementioned findings seem to reflect association between increased severity of tumor-associated inflammation and worse oncologic outcomes. However, neutrophil-lymphocyte ratio threshold should be further investigated to identify an optimal value that can be agreed upon by clinicians, and that can be relied upon to deliver clinical utility.

Monocytes are progenitors of macrophages, which are the key mediators of the immune system. Thus, circulating monocyte count is regarded as a surrogate marker for tumor-associated macrophages (TAMs). TAMs are classified into M1 and M2 types, with each having different roles in human and cancer cells. Activated M1 promotes anti-tumor response to eliminate tumor cells. In contrast, M2 suppresses adaptive immune response, and produces vascular endothelial growth factor (VEGF). TAMs were reported to have poor prognostic impact on survival in various types of malignancy, including lymphoma, lung adenocarcinoma, endometrial carcinoma, and ovarian carcinoma [24, 25]. A previous study in lung adenocarcinoma reported monocyte count > 430/mm^3^ to be an independent predictor of recurrence-free survival and OS (HR = 1.765, 95% CI: 1.071–2.910, *P* = 0.0258, and HR = 4.339, 95% CI: 2.032–9.263, *P* < 0.001, respectively) [24]. A study in 141 patients with stage I-IV endometrial carcinoma found monocyte count > 500/mm^3^ to be an independent predictor of decreased survival time after recurrence/progression (HR = 3.12, 95% CI: 1.52–6.67, *P* < 0.001) [25]. Subsequent study in 541 patients with stage I-IV endometrial carcinoma reported monocytes > 700/mm^3^ to be significantly associated with deep myometrial invasion, pelvic lymph node metastasis, and advanced stage. That study also found monocytes > 700/mm^3^ to be independently associated with decreased disease-free survival (HR = 1.74, 95% CI: 1.02–2.96, *P* = 0.041) and decreased OS (HR = 2.63, 95% CI: 1.37–5.05, *P* = 0.004) [26]. An earlier study in 788 patients with stage IB1-IVA squamous cell carcinoma of the cervix reported high monocyte count as a poor prognostic factor for PFS and OS (HR =5.37, 95% CI: 1.594–18.10, *P* = 0.007, and HR = 3.97, 95% CI: 1.076–14.61, *P* = 0.038, respectively) [27]. The current study observed a contrary finding, that elevated monocyte count was not an adverse prognostic factor for PFS or OS.

Paraneoplastic thrombocytosis was driven by IL-6 and hepatic thrombopoietin, both of which stimulated tumor cell proliferation and migration; however, the mechanism is not clearly understood [28]. Previous studies in cancer patients showed that a platelet count > 400,000/mm^3^ correlated with advanced stage of disease and decreased 5-year OS (odds ratio = 2.70, 95% CI: 2.03–3.61) [29]. The impact of pretreatment platelet counts in early stage cervical cancer is still being debated [30]. A study in presurgical stage IB1 cervical cancer, and a GOG study in preradiated cervical cancer, revealed that increased platelet count before treatment initiation was an independent predictor of poor survival [31, 32]. In contrast, multivariate analysis in 3 studies in early stage cervical cancer found that a higher preoperative platelet count did not significantly impact PFS or OS [33–35]. The current study in stage IVB, persistent, or recurrence cervical cancer found platelet count > 400,000/mm^3^ to be an independent predictor of short OS. This finding was similar to the findings from studies in patients with epithelial ovarian carcinoma and endometrial cancer [28, 36, 37].

The strength of the current study is that common laboratory measurements were used before initiation of chemotherapy, which means that the proposed ≥3.6 neutrophil-lymphocyte ratio parameter can be used in routine practice, even in limited-resource settings. To the best of our knowledge, this is the first clinical study in the predictive effects of hematologic parameters in cervical cancer patients treated by chemotherapy. These results may be applied for use in a clinical application as a biomarker for predicting chemotherapy response, cancer recurrence, or progression after complete treatment.

This study also has some mentionable limitations. The first is its, uncontrolled non-randomized retrospective design. Second, the broad range of histologic subtypes and chemotherapeutic regimens included in this study are potential confounders. However, all histologic subtypes mentioned in this study were found in the real practice and treated with the standard treatment guideline, and all of the chemotherapeutic regimens described in this study were reported to be effective in the treatment cervical cancer. Third and last, the number of hematopoietic cell counts may not represent their functions, and each cut-off value used in this study requires validation in other populations.

Future studies should investigate the predictive effects of neutrophil-lymphocyte ratio and absolute neutrophil and monocyte counts for predicting overall response and survival relative to both appropriate cut-off values, and comparison between baseline values and nadir in first cycle or decrease in subsequent cycles of chemotherapy. Second, a prospective multicenter study should be conducted to validate the Glasgow prognostic inflammatory score in this patient population, and to establish a population specific score, particularly in high incidence countries. Third, increased understanding of intratumoral stroma and tumor islets infiltrating leukocytes would increase our understanding of the powerful impact of hematologic parameters on cancer survival. A race/continent-specific prognostic model/score using this biological information may serve as an appropriate stratification factor before treatment allocation to chemotherapy or palliative treatment. Another clinical application is as a biomarker for chemotherapy response, or predicting cancer recurrence or for predicting cancer progression after complete treatment. A novel therapeutic modality using a leukocyte-mediated approach may obtain better treatment outcomes.

## Conclusions

Neutrophil-lymphocyte ratio ≥ 3.6 was identified as an independent predictor of poor oncologic outcomes relative to overall response rate, PFS and OS.

## Abbreviations

BMI: Body mass index
CI: Confidence interval
FIGO: The International Federation of Gynecology and Obstetrics
G-CSF: Granulocyte colony-stimulating factor
GOG: Gynecologic Oncology Group
HR: Hazard ratio
IL: Interleukin
IQR: Interquartile range
OS: Overall survival
PFS: Progression free survival
RECIST: Response evaluation criteria in solid tumor
SD: Standard deviation
TAMS: Tumor associated macrophages
VEGF: Vascular endothelial growth factor
WHO: World Health Organization
